# Longevity and Culling Reasons in Dairy Herds in Southern Brazil

**DOI:** 10.3390/ani15152232

**Published:** 2025-07-29

**Authors:** Rodrigo de Almeida, Sidneia de Paula, Marianna Marinho Marquetti, Milaine Poczynek, Delma Fabíola Ferreira da Silva, Rodrigo Barros Navarro, Altair Antonio Valloto, José Augusto Horst, Victor Breno Pedrosa

**Affiliations:** 1 Programa de Pós-Graduação em Zootecnia—PPGZ, Universidade Federal do Paraná—UFPR, Rua dos Funcionários, 1540, Curitiba 80035-050, PR, Brazil; sidneia.paula@gmail.com (S.d.P.); marianna.marquetti@ufpr.br (M.M.M.); milainepoc@gmail.com (M.P.); delmaffsilva@idr.pr.gov.br (D.F.F.d.S.); 2 Capal Cooperativa Agroindustrial, Rua Saladino de Castro, 1375, Arapoti 84990-000, PR, Brazil; rodrigo@capal.coop.br; 3 Associação Paranaense dos Criadores de Bovinos da Raça Holandesa, Rua Professor Francisco Dranka, 608, Curitiba 81200-404, PR, Brazil; altair@apcbrh.com.br (A.A.V.); horst@apcbrh.com.br (J.A.H.); 4 Neogen Corporation, 4131 N 48th St., Lincoln, NE 68504, USA; vbpedrosa@gmail.com

**Keywords:** dairy cattle, herd life, herd turnover rate, replacement

## Abstract

This study aimed to evaluate cow longevity and identify the main culling reasons in dairy herds in Southern Brazil. Two data sets from 26 predominantly confined Holstein herds were analyzed over a 10-year period (2007–2016). The first included 11,150 cows that were culled, died, or sold, and the second comprised 636,739 cows for demographic analysis. The average annual culling rate was 24.2%, mainly due to reproductive disorders (34.0%), mastitis/high somatic cell count (20.4%), and feet and leg problems (17.9%). Involuntary causes represented 89.5% of all culling. The death rate averaged 3.8%, with the most frequent causes being unknown (27.3%), other reasons (25.6%), tick fever (10.2%), and accidents/injuries (10.0%). Larger herds had higher culling rates than smaller ones (26.2% vs. 22.8%; *p* = 0.04), as did higher-producing herds compared to lower-producing ones (25.7% vs. 22.0%; *p* = 0.02). Cows with ≥5 calvings were culled more often (*p* < 0.01) than those in earlier lactations. Culling was lowest (*p* < 0.02) in spring and highest (*p* < 0.01) during early (0–60 d) and late (>420 d) lactation. Herds with a higher proportion of older cows had slightly lower milk yields (*p* < 0.01), indicating longevity does not always enhance productivity.

## Simple Summary

Culling, or the removal of cows from the herd, is an important part of managing dairy farms. Understanding why cows are removed and how long they stay in the herd helps improve animal welfare and farm productivity. In this study, we looked at data from 26 dairy farms in Southern Brazil over a 10-year period. We found that nearly one in four cows was removed each year, mostly due to health problems like reproductive failure, udder infections, and leg or hoof conditions. Most cows were culled for reasons that farmers could not control. Larger and more productive farms had higher culling rates. Older cows, especially those with five or more calvings, were more likely to be removed. Spring had the lowest culling rates, while the beginning and the end of lactation were the riskiest periods. Interestingly, farms with more older cows produced slightly less milk, suggesting that simply keeping cows longer does not always lead to maximum profitability. These findings can help farmers make better decisions to improve both cow welfare and farm efficiency.

## 1. Introduction

The culling rate of a dairy farm is affected by several factors like the incidence of diseases, size and productivity of the herd, replacement heifer’s availability, milk prices, and genetic selection goals [1]. Although it is well established that dairy cows only achieve the highest productivity in the 3rd lactation or even greater [2], replacing multiparous cows with young and genetic-improved heifers can increase the farm’s profitability. The daily risk of culling is typically greater in early lactation, and it is also well-known that culling cows during the onset of lactation may cause relevant economic losses [3].

More recently, ref. [4] discussed the importance of increasing the productive lifespan of dairy cows, considering sustainability and profitability factors. However, it is essential to remember that a longer productive life is not necessarily equivalent to higher profitability [1]. On the other hand, a shortened productive life due to health issues will always mean lower profitability and sustainability as well [1]. It is currently estimated that it takes around 1.5 lactations for a dairy heifer to break even the investment made in her rearing [2,3].

The productive lifespan of dairy cows may be defined as the time from first calving to death and it is short compared to their natural life expectancy. Although their natural life expectancy is approximately 15–20 years, the productive lifespan of average cows is between 2.5 and 4 years in most developed dairy countries, which brings their total lifespan from birth to death between 4.5 and 6 years [4]. High-producing dairy herds in Brazil are showing a high proportion of primiparous (>40%) and a low proportion of adult cows (<30%) among the lactating animals, raising questions about the welfare and ethical use of dairy cattle [5].

Culling is the departure of cows from the herd because of sale, slaughter, salvage, or death, and it has been classified as voluntary or involuntary or as an economic or biological culling [6,7]. Involuntary culling implies that the producer was forced to remove cows that were culled due to disease, infertility, injury, or death. Voluntary culling happens when the farmer makes the decision to choose which cows will be removed from the herd, primarily due to low production. Culling risks are affected by physiological processes, such as calving, lactation, negative energy balance, reproduction, and aging [8]. Housing conditions and the management of animals also have a significant impact on the risk of culling, as well as the environment and the availability of replacement animals [4]. Even if some cows leave the herd for involuntary reasons, management decisions largely determine the average productive life of dairy cattle [7]. Furthermore, economically important performance indicators such as reproduction, production, health, and young-stock performance are essential for farm-level evaluation [9].

Previous studies have documented that longer calving intervals [10], predominance of the Holstein breed [11], and larger herds [12] are all factors associated with increased culling rates. Countries with intensive production systems, as practiced in USA and Israel, have annual culling rates of around 30–38% [13].

As mentioned before, the culling rate and the reasons for removal vary according to country or region, production system, and management decisions. Despite the importance of culling decisions on dairy farm profitability, there is no data (as far as we know) about the reasons that farmers apply when they decide to cull a cow in Brazilian dairy farms. When comparing the Brazilian dairy farms with herds from other traditional dairy countries, one initial difference is the fact that most Brazilian herds are still growing, and in this case, dairy owners typically keep lower-producing cows to fill the new buildings as fast as possible. Another notable difference in the Brazilian dairy industry from other traditional dairy countries is the possibility to use rbST on lactating dairy cows, which may impact the productive life of a dairy cow, and maybe it is one of the explanations for the low proportion of cows being culled due to low production. Unfortunately, it is not easy to account for rbST in the analysis because its adoption is very heterogeneous within time and among herds.

Our initial hypothesis was that involuntary culling (due to factors like health issues or reproductive problems) is more prevalent than voluntary culling (due to low production) in dairy herds. Additionally, it proposes that larger, higher-producing herds experience higher culling rates than smaller, lower-producing herds, and that parity, stage of lactation, and the time of calving and culling can influence culling rates. So, the first goal of this study was to identify the main culling and death reasons of Brazilian dairy cows, obtained primarily by a data set from a Dairy Herd Improvement (DHI) service and detailed by personal annual interviews over a 10-year period. The second objective was to evaluate the effects of productivity and herd size on annual culling rates. Finally, the third goal of this study was to measure the impact of parity, lactation stage, calving season, and culling season on the culling rates.

## 2. Materials and Methods

### 2.1. Data Source, Interviews, and Editing

Initially, data sets from the 26 monitored farms were obtained from Associação Paranaense de Criadores de Bovinos da Raça Holandesa (APCBRH), the Holstein Breed Association of Paraná State, Southern Brazil. Although Paraná State is the second largest milk producer in Brazil, it has the most important DHI organization in Brazil, with almost 70% of all Brazilian official milk-recorded cows. Annually, APCBRH provided us with a data set containing all cows that left their herds in the previous year. After 10 years of monitoring these 26 herds, from 1 January 2007, through 31 December 2016, we had reached a total of 11,150 lactating and dry cows sold for dairy purposes, culled, or died on the farm.

All 26 dairy herds monitored were comprised almost exclusively of Holstein cows, in confined dairy systems (free-stall or compost barn), and they were located in the Arapoti county, Paraná State, Southern Brazil, 24°8′43″ S 49°49′8″ W. The climate in Arapoti is classified as a subtropical climate. Summer is warm and humid, with temperatures averaging around 26–29 °C. Winter is cool and dry, with temperatures fluctuating between 13 and 22 °C. Spring and fall are transitional seasons, with temperatures varying between 14 and 23 °C.

Each cow that left the herd was initially categorized as a dairy sale, slaughter (culling), or death, following the recommendations of [7]. These 26 herds with similar management and milked 2 or 3x/day, were monitored in a 10-year period. Herd owners or herd managers were personally interviewed annually, typically on February or March in the following year, by a graduate student from Universidade Federal do Paraná (UFPR), to describe the primary and the secondary reasons why cows were culled in the previous year, as well as the exact date the cows left the herd.

The codes used to describe the culling reasons were divided into 6 groups: (1) diseases: udder health (mastitis and high somatic cell count—SCC), feet and leg problems (claw disease or leg problems), displaced abomasum, metabolic diseases (milk fever, ketosis, bloat), tick fever (anaplasmosis and babesiosis), leukosis, accident/injury, hardware, heart problems, intoxication or poisoning; (2) reproduction: low fertility (not pregnant and abortion or pregnancy losses); (3) production: low production, poor milking ability; (4) physical: deformed teats or udder, feet and legs conformation problems, low type classification scores; (5) high age (aging); (6) others: poor temperament and unspecified reasons or diseases, as described in Table 1.

A second data set was obtained from APCBRH containing data from the same 26 herds in the same 10-year period (2007–2016) with the following information: monthly average milk yield, number of lactating + dry cows with one, two, and three or more calvings, days in milk (DIM), % milk fat, % milk protein, the average age in months, and the average age at first calving (Table 2). From this data set, the parameter monthly proportion of cows with 3 or greater parities was estimated.

A third data set was provided for longevity and demography analysis, which included 636,739 lactating and dry cows, from the same 26 herds. Annual culling rates for each herd were estimated by dividing the number of cows culled over a year by the population at risk of being culled over the same period, where the population at risk was determined by monitoring all cows in the herd and counting the number of culls in that cohort [6,7]. Herds were categorized based on size, with the following criteria: small (≤150 lactating and dry cows), medium (151–250 lactating and dry cows), and large (>250 lactating and dry cows). Herds were also categorized based on productivity; low-producing (<9100 kg/year), intermediate-producing (9100–9700 kg/year), and high-producing (>9700 kg/year), with the concern of having approximately a third of the herds on each class.

### 2.2. Statistical Analysis

Data editing and analysis was performed with version 9.4 of SAS software (SAS Institute Inc., Cary, NC, USA). Simple Pearson correlations among culling and milk yield, milk fat, and protein contents were estimated by CORR procedure and the GLM procedure was used to estimate the effects of year, herd size, and herd productivity on the variable culling rate.

Pearson correlations between the proportion of cows with 3 and greater parities and year, DIM, age, milk yield, milk fat, and milk protein contents were also estimated by the CORR procedure. The GLM procedure was adopted to evaluate the effects of year and month on the proportion of cows with ≥3 parities.

Two distinct modeling approaches were used, both analyzing the same dependent variable: culling rate (percentage of cows culled). The first analysis aimed to evaluate the effects of herd-level traits (herd size and milk production level) on overall culling rate, using herd–year combinations as observational units (see Table 3). The second analysis focused on the influence of cow-level factors (parity, days in milk, calving season, and culling season) on the proportion of culling within each category, including herd as a random effect to account for clustering (see Table 4). These two models were applied independently but used the same data set and outcome variable.

In order to verify the potential impact of herd size and productivity on the culling rate, data was analyzed with Tukey’s 95% test under the MIXED procedure of SAS. Initially, the 26 herds and the 10-year study period were combined in herd*year classes and categorized by two variables: productivity; high-producing herds (>9700 kg/year; *n* = 66), herds with intermediate yields (9100 to 9700 kg/year; *n* = 61), and low-producing herds (<9100 kg/year; *n* = 58), and herd size; large (>250 animals; *n* = 60), medium (150 to 250 animals; *n* = 73), and small (<150 animals; *n* = 60) herds, adding lactating and dry cows. The number of herd*year classes was lower than the potential 260 classes (26 herds x 10 years) because a few herds left or entered the dairy activity during the 10-year study interval.

Lastly, the proportion of culling (%) was tested for four different variables: five parity classes (1, 2, 3, 4, and 5+); eight DIM categories (0 to 60 d, 61 to 120 d, 121 to 180 d, 181 to 240 d, 241 to 300 d, 301 to 360 d, 361 to 420 d, and >420 d); four calving seasons (summer, fall, winter, and spring), and four culling seasons (summer, fall, winter, and spring). For this analysis, the unit of observation was herd, included in the model as a random effect. Specifically, the proportion of cows culled within each category (e.g., parity class, days in milk group, calving season, or culling season) was calculated separately for each of the 26 herds. As a result, each class level within these categorical variables includes 26 observations, corresponding to the 26 herd-level means. The least squares means shown in Table 4 represent the estimated average within-category proportion of cows culled. These values are not cumulative nor directly comparable across unrelated factor groups (e.g., comparing DIM classes with seasons), as each factor was modeled independently. Statistical differences were reported when *p* value < 0.05.

## 3. Results

A total of 11,150 cows left the 26 monitored herds; 9065 cows were culled, 1592 died, and 493 were sold for dairy purposes, resulting in 24.2% average culling rate and 3.8% death rate, as demonstrated in Table 1.

**Figure 1:**
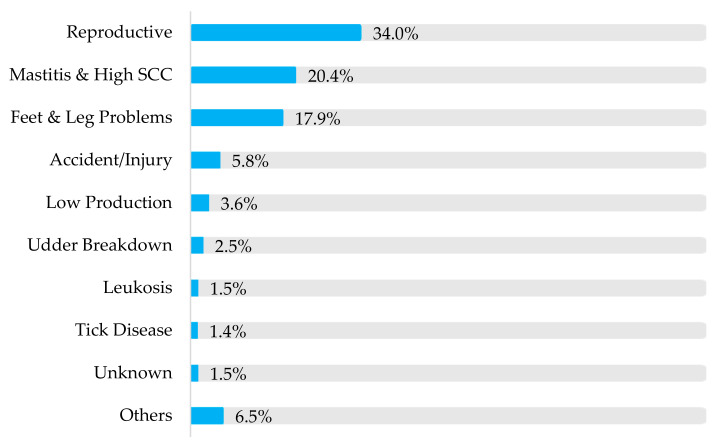
Percentage distribution of culling in commercial dairy herds in Arapoti, Paraná, Brazil, based on records collected from 2007 to 2016.

Involuntary culls were 89.5%, and the voluntary culls were 10.5%, as shown in Figure 1.

Culling rates for reproductive failure were 34.0%, mastitis and high SCC at 20.4%, and feet and leg problems at 17.9%, which are the three, main culling reason groups.

**Figure 2:**
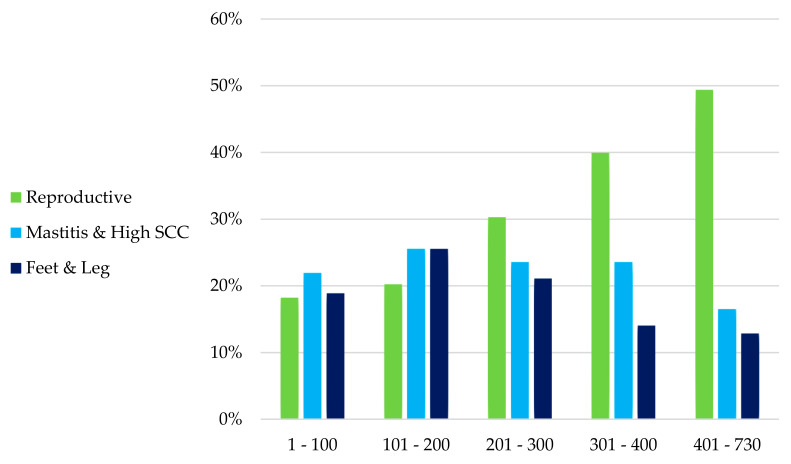
Distribution of main culling reasons according to stage of lactation in Arapoti herds from 2007 to 2016.

The percentage of culling by reproductive failure, mastitis, and high SCC, and feet and leg problems varied during lactation (Figure 2). Mastitis and high SCC and feet and leg problems culls were well distributed along the lactation but culling due to reproductive failure was more frequent at the end of lactation.

**Figure 3:**
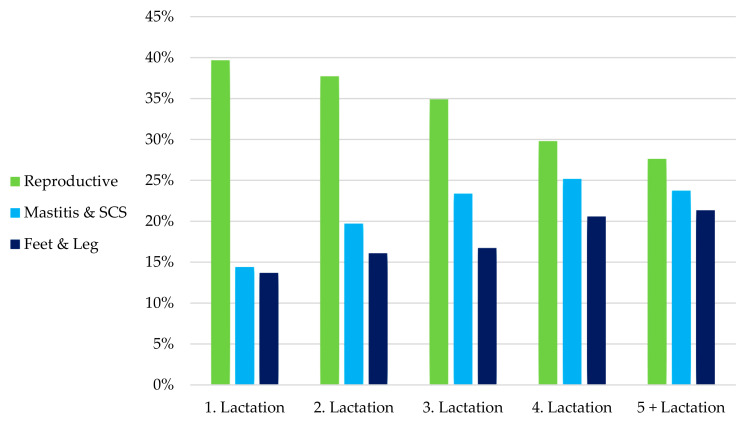
Distribution of main culling reasons according to parity order in dairy herds located in Arapoti, Paraná, Brazil, from 2007 to 2016.

The main culling reasons also differed among parities (Table 2; Figure 3). It seems that first-parity cows are more frequently discarded due to reproductive failure, but older cows left the herds by the three main reasons equally.

### 3.1. Herd Milk Production Level and Herd Size Categories

The farms that had average production per lactation above 9700 kg, and farms between 9700 and 9100 kg, categorized as high and moderate milk production herds, had annual culling rates slightly above 25%. Meanwhile, the group of farms categorized as the lower-producing ones, with average production less than 9100 kg per lactation, culled proportionally less cows (22%; *p* < 0.01) (Table 3).

Culling rates were greater (*p* < 0.04) in larger herds (up to 250 cows) than in smaller herds (up to 150 cows); 26.2 vs. 22.8%, respectively (Table 3).

No differences in culling rates were found among cows from 1st to 4th parities; however, cows with five or greater parities had greater culling rates than cows with two and four parities (*p* < 0.01), as shown in Table 4.

Noticeable differences (*p* < 0.01) in culling rates among classes of days in milk were observed, especially higher culling rates in the first (0–60 DIM) and in the last (>420 DIM) classes, and lower culling rates in the intermediate classes, among 60 and 420 DIM.

Cows that calved during spring season showed a lower (*p* < 0.01) culling rate than cows that calved in the remaining seasons. Similarly, the lowest culling rate occurred during spring season (*p* = 0.02; Table 4), compared with other seasons.

### 3.2. Longevity and Death

As expected, the effects of herd and year were significant (*p* < 0.01) in the proportion of cows with three and greater parities during the evaluated 10-year period. The average percentage of cows with ≥3 parities was 37.0% and the average cow age was 46.1 months.

The average death rate was 3.8%, and the main reasons were unknown (27.3%), followed by tick fever (10.2%), accidents and injuries (10.0%), dystocia (6.7%), mastitis (5.4%), death due to heart problems (4.1%), metabolic diseases like milk fever, clinical ketosis, and bloat (3.5%), death due to feet and legs problems (2.8%), displaced abomasum (2.4%), leukosis (2.0%), and other reasons (lower than 2%; 25.6%).

## 4. Discussion

The primary objective of this study was to identify the main culling and death reasons of dairy cows in a 10-year period (2007–2016), as well as to estimate the culling rates and their associations with milk yield, herd size, parity, DIM, calving, and culling seasons. Another measure of interest was to analyze the proportion of cows with three or higher parities as a longevity trait in dairy herds located in Southern Brazil.

Considering that the expected increase in the world population until 2067 reaches 10.4 billion people, dairy herds will need to balance profitability with sustainability to meet the demands for dairy products [14]. For this reason, herd replacement and culling rates are gaining increasing relevance. In addition, there is a perception that a minority of dairy cows in commercial herds are achieving maturity, and this fact can impact how the general public and dairy consumers see the dairy industry.

The culling rates here observed were similar to dairy herds in Southern Iran [15] and the Netherlands [16], but they were lower than the annual culling rates reported in the United States [17,18] and Canada.

In the present study, like most other references around the world [12,15,18], the most prevalent culling reason of dairy cows was infertility or failure to conceive or remain pregnant, because pregnancy greatly reduces the risk of culling [19]. Cows that conceive more readily than their herd mates remain in the herd longer [20]. In the same manner, a cow that has more difficulty conceiving [8,18] had a four times greater risk of culling compared with pregnant cows [17]. Thus, the cost per extra days open increases when cows are on average longer on milk [21]; consequently, the culling risk of non-pregnant cows is even more critical in mid-lactation animals.

Other reproductive reasons are associated with culling, such as calving ease, calf size, number of inseminations, days from calving to first insemination, and days from calving to conception, also known as days open. All these reproductive factors impact the functional productive life of dairy cows [22]. Both the reproductive management as well as the genetic selection for reproductive traits can be improved, reducing the number of cows that are removed from commercial herds for reproductive failure [1,23,24].

Contrary to other studies conducted previously, where the second main reason for culling was low production [24], in the current study, mastitis and high SCC were the second most prevalent culling reasons. It is well known that mastitis affects dairy production reducing milk yield and its quality, as well as the yield and the shelf life of dairy products [25], being one of the most common and costly diseases in dairy cattle [4].

Although clinical mastitis can be observed throughout the entire lactation [8], mastitis incidence is typically greater in early lactation [26]. When a cow has subclinical mastitis, she will produce less milk and have an elevated SCC, so farms that have higher SCC have a greater culling rate [16]. Mastitis is a relevant disease in all herd size categories and regions worldwide, and it is considered the disease that most influences culling in dairy herds [26,27].

The welfare of dairy cattle is already an area of increasing demand, and the research in this area is expected to continue gaining prominence, aiming to improve facilities and to provide more comfort to the animals [14]. Feet and leg problems, as lameness, are widely recognized as the most serious signs that the welfare of the animals is poor [28]. It is also known that locomotion problems negatively impact reproductive efficiency and milk yield of dairy cows, increasing the hazard of culling and death [29]. Lame cows will eat less and reduce estrus expression or avoid being mounted by other cows, compromising reproductive performance [4]. Poor conditions in the facilities such as hot and wet environments, concrete floors, or low comfort of the lying stalls can aggravate the scenario [30]. Reproductive disorders, mastitis and high SCC, and feet and leg problems in dairy cattle are often linked to poor management practices, particularly during the transition period, and environmental factors, including bacterial infections.

Our findings are in agreement with the association between culling risk and DIM, with culls and deaths being more common at the very beginning or at the end of lactation [8,18]. In early lactation, dairy cows typically show negative energy balance, inflammation, and oxidative stress, which increase the risk of and culling rate until 60 DIM [31,32,33]. Culling a cow at the beginning of lactation most probably is an involuntary decision and compromise profitability. The risk of culling increases according to the stage of lactation in cows that fail to get pregnant as shown in our study (22.3% in cows with DIM higher than 420 d).

The voluntary culls, mainly due to low production, is an economic decision about cows producing below a certain threshold that will leave the herd and be replaced by a higher-producing cow. In the last decades, the culling rate attributed to low milk production had decreased from 17% to 4% [34], a similar rate detected in the present study (3.6%). This trend of a few cows being discarded due to low production was also found in another study conducted in Brazil, with Holstein and Girolando cows [35]. Higher milk production was associated with a lower relative risk of culling [8]; however, high-producing cows are more prone to diseases, injury, and infertility [36] than low-producing ones and they have higher culling rate than low-producing cows, as shown in our study (25.7 vs. 22.0%).

In larger herds, more cows were culled than smaller herds (26.2% vs. 22.8%), either because they are already stabilized or because they adopt a higher herd replacement rate, similar findings described in other studies in North America [12,16,18]. However, other references showed that herd size did not have a clear association with reproductive efficiency [37]. Herds in expansion had a negative effect on culling, particularly the voluntary culling [18], and tended to postpone the decision of culling a cow, either for emotional reasons, where the animals are more than just a number in the herd, or for the desire to fill the property. This is probably another reason why so few cows in the present study were discarded due to low production.

A primary goal of dairy producers is to keep culling numbers in the first lactation as low as possible [18], because this premature culling seems to have a negative effect on profitability [38]. Herds with a longer average age expect benefits from improving lifetime productivity. Extending productive lifetime will increase milk production and profitability per cow because a first lactation cow may produce around 80% of a mature cow [6], and healthy cows reach full maturity approximately from the third to the fifth lactation, when they produce more milk. On the other hand, replacing mid-lactation and no pregnant multiparous cows for a genetically improved heifer is a common management decision.

It was expected that cows that produce more milk fat and protein contents were less likely to be culled [18], but we found no correlation among them in our study. The lack of association in our study may be due to the fact that the herds being monitored in the present study have very similar production systems. Another possible reason for this lack of association between culling and milk components is the fact that this data set was generated between 2007 and 2016, a period in which the milk payment system was not fully adopted in the region.

The effects of calving and culling seasons were very similar, and they both showed that the best calving and culling season, with the lowest risk of culling, was spring. This finding is not unexpected because in spring (from September to December in the Southern Hemisphere), we have better milk prices and the highest milk productivity in the year. Also, dairy cows that calved in spring had the opportunity to pass through their transition period in winter, with lower temperatures and no heat stress [38]. Heat stress during the dry period decreased milk production in the subsequent lactation [39] and cows dried in hot months had an increased number of breeding, days to first breeding, and days to pregnancy diagnosis during the first 150 DIM in the subsequent lactation relative to those dried-off in the cooler months [21,40].

In the present study, a large proportion of cows had no specific cause of death, 27.3% were unknown, and 40.4% other reasons, while the United States data reported 20% of unknown reasons [41], suggesting that Brazilian veterinarians who attend dairy farms should do more necropsy to identify the reasons of death in dairies. Dairy industry intensification may be related to the risk factors for cow culling and mortality and about 50% of cow death losses are attributable to causes that could be mitigated with proper management [41]. Among the management practices which contribute to reducing the mortality rate and herd exits on dairy farms are improvements in reproductive efficiency, cow comfort, health care, transition period, and heat stress mitigation [3].

Mortality rates in dairy cows were lower [10] as expected, and the result is probably due to the lack of information or methods of analysis. The third highest death reason was tick disease, which is not present in cattle raised in Europe and North America. Nonetheless, part of our results agrees with the data from the Canadian DHI Valacta (2016), because the proportion of cows with three and greater parities found here (37.1%) was very similar with the average number found in Quebec dairy herds (39.1%), suggesting that our herds also prioritize longevity. Another possibility is that in the 10-year period of our study, satisfactory milk prices may have limited the number of cows being discarded.

## 5. Conclusions

The most important culling reasons were reproductive disorders, followed by mastitis and high SCC and feet and leg problems, like other high milk-producing countries around the world. The main difference was our low proportion of cows being discarded for low production and our high rate of involuntary culling. It was also observed that larger and higher-producing herds have higher culling rates. To improve the longevity of dairy cows and support dairy farmers, focusing on key areas like transition period health, mastitis, lameness, and pregnancy rate is crucial.

## Figures and Tables

**Table 1:** Total number of cows assigned to each culling reason for diseases/health, reproduction, production, physical, aging, and others/unknown categories, and distribution between voluntary (VC) and involuntary (IC) culling.

Culling Category	Culling Reason	VC/IC	Total (*n*)	Percentage
**Diseases/Health**	Mastitis	IC	1373	15.1%
	High SCC	IC	483	5.3%
	Feet and legs	IC	1626	17.9%
	Displaced abomasum	IC	89	1.0%
	Metabolic diseases (milk fever, ketosis, fatty liver, etc.)	IC	94	1.0%
	Anaplasmosis/babesiosis	IC	124	1.4%
	Leukosis	VC	140	1.5%
	Accident/injury	IC	525	5.8%
	Hardware	IC	21	0.2%
	Intoxication and/or poisoning	IC	14	0.2%
	Heart problems	IC	36	0.4%
**Reproduction**	Abortion/reabsorption	IC	268	3.0%
	Nonpregnant	IC	2732	30.1%
	Dystocia	IC	80	0.9%
**Production**	Low production	VC	329	3.6%
**Physical**	Slow milking speed	VC	11	0.1%
	Udder breakdown	VC	224	2.5%
	Low feet and legs score	VC	69	0.8%
	Bad temperament	VC	17	0.2%
	Low classification final score	VC	13	0.1%
**High age**	Aging	VC	71	0.8%
**Others**	Others	-	589	6.5%
	Unknown	-	137	1.5%
**Total**			**9.065**	**100.0%**

-: "Others" culling reasons could be voluntary or involuntary.

**Table 2:** Descriptive statistics (means ± SD) of dairy herds located in Southern Brazil, based on production and herd structure data collected from 2007 to 2016.

Variable	2007	2008	2009	2010	2011	2012	2013	2014	2015	2016	Average
Number of herds	19	19	21	21	16	17	17	19	21	23	19.3
Herd size (cows)	176 ± 83	183 ± 84	186 ± 82	186 ± 81	187 ± 91	190 ± 100	192 ± 106	199 ± 111	207 ± 121	201 ± 130	191 ± 101
% parity 1	35.8	36.0	34.0	32.7	33.4	35.3	31.6	32.2	37.6	36.9	34.9
% parity 2	28.9	27.8	28.3	27.3	26.6	28.2	27.4	27.5	27.3	28.5	28.1
% parity ≥ 3	34.7	35.7	37.3	39.6	39.7	39.8	36.1	34.6	35.0	34.5	37.0
Age (mo)	47.7 ± 6.1	47.4 ± 5.7	47.1 ± 5.1	47.5 ± 4.9	47.5 ± 6.6	46.9 ± 7.4	46.0 ± 6.6	44.6 ± 5.9	43.3 ± 5.8	43.3 ± 4.7	46.1 ± 6.2
Milk yield (kg/d)	29.6 ± 3.4	29.7 ± 3.3	30.1 ± 3.6	30.4 ± 3.6	31.5 ± 3.8	31.0 ± 3.5	30.7 ± 3.7	30.2 ± 4.1	30.0 ± 3.9	29.8 ± 3.7	30.3 ± 3.7
% Milk Fat	3.30 ± 0.29	3.19 ± 0.33	3.32 ± 0.26	3.30 ± 0.28	3.28 ± 0.29	3.44 ± 0.28	3.46 ± 0.34	3.54 ± 0.34	3.60 ± 0.34	3.60 ± 0.34	3.41 ± 0.34
% Milk Protein	3.10 ± 0.11	3.10 ± 0.09	3.12 ± 0.10	3.16 ± 0.10	3.17 ± 0.10	3.18 ± 0.11	3.18 ± 0.11	3.20 ± 0.11	3.20 ± 0.09	3.23 ± 0.11	3.17 ± 0.11
Age at first calving	27.1 ± 1.8	27.4 ± 2.3	28.1 ± 3.3	28.0 ± 3.4	27.0 ± 2.8	27.1 ± 2.6	26.4 ± 2.2	26.3 ± 2.5	26.3 ± 2.2	26.5 ± 2.6	27.0 ± 2.7
DIM	191 ± 23	187 ± 21	187 ± 20	196 ± 23	194 ± 28	195 ± 25	193 ± 23	192 ± 30	190 ± 23	187 ± 25	191 ± 25

**Table 3:** Culling rates (means ± standard errors) in Arapoti, Paraná, Brazil, dairy herds according to milk yield levels and herd size category.

Group	Subgroup	Culling Rate (%) ± S.E.	*p* Value
Milk Production Level	Low (<9100 kg; *n* = 53)	22.0 b ± 1.0	0.02
	Moderate (9100–9700 kg; *n* = 83)	25.2 a ± 0.7	
	High (>9700 kg; *n* = 49)	25.7 a ± 0.9	
Herd Size	Small (<150; *n* = 59)	22.8 b ± 1.1	0.04
	Medium (150–250; *n* = 73)	24.3 ab ± 0.7	
	Large (>250; *n* = 60)	26.2 a ± 0.8	

Means followed by common letters in the column do not differ statistically at 0.05 significance level by Tukey test.

**Table 4:** Culling rates (means ± standard errors) in dairy cows stratified by parity order, stage of lactation (days in milk), calving season, and season in which culling occurred.

Group	Subgroup	Culling Rate% ± S.E.	*p*-Value
Parity	1	20.0 ab ± 0.7	<0.01
	2	19.2 b ± 1.0	
	3	19.9 ab ± 0.7	
	4	17.8 b ± 0.7	
	≥5	23.2 a ± 1.2	
Days in milk	0–60	16.8 b ± 1.1	<0.01
	61–120	11.9 c ± 0.6	
	121–180	10.4 c ± 0.5	
	181–240	10.0 c ± 0.4	
	241–300	9.6 c ± 0.4	
	301–360	10.2 c ± 0.5	
	361–420	8.9 c ± 0.6	
	>420	22.3 a ± 1.3	
Calving season	Summer	25.6 a ± 0.6	<0.01
	Fall	26.6 a ± 0.5	
	Winter	26.5 a ± 0.5	
	Spring	21.3 b ± 0.8	
Culling season	Summer	26.1 ab ± 1.1	0.02
	Fall	26.8 a ± 0.9	
	Winter	26.1 ab ± 0.8	
	Spring	23.0 b ± 0.8	

Means followed by common letters in the column do not differ statistically at 0.05 significance level by Tukey test.

## Data Availability

The original contributions presented in this study are included in the article. Further inquiries can be directed to the corresponding author.

## References

[1] De Vries A. (2020). Symposium review: Why revisit dairy cattle productive lifespan?. J. Dairy Sci..

[2] NASEM (2021). Nutrient Requirements of Dairy Cattle.

[3] De Vries A. (2006). Economic value of pregnancy in dairy cattle. J. Dairy Sci..

[4] De Vries A., Marcondes M.I. (2020). Review: Overview of factors affecting productive lifespan of dairy cows. Animal.

[5] Bueno G.A.Z., Marquetti M.M., Horst J.A., Valloto A.A., Almeida R. A demographic study of milk-recorded dairy cows in Paraná State. Proceedings of the 58th Brazilian Society of Animal Science Annual Meeting.

[6] Dohoo I.R., Dijkhuizeu A.A. (1993). Techniques involved in making dairy cow culling decisions. Compend. Contin. Educ. Pract. Vet..

[7] Fetrow J., Nordlund K.V., Norman H.D. (2006). Invited Review: Culling: Nomenclature, definitions, and recommendations. J. Dairy Sci..

[8] Pinedo P.J., De Vries A., Webb D.W. (2010). Dynamics of culling risk with disposal codes reported by Dairy Herd Improvement dairy herds. J. Dairy Sci..

[9] Schuster J.C., Barkema H.W., De Vries A., Kelton D.F., Orsel K. (2020). Invited review: Academic and applied approach to evaluating longevity in dairy cows. J. Dairy Sci..

[10] Alvåsen K., Roth A., Jansson Mörk M., Sandgren H., Thomsen P.T., Emanuelson U. (2014). Farm characteristics related to on-farm cow mortality in dairy herds: A questionnaire study. Animal.

[11] Thomsen P.T., Kjeldsen A.M., Sørensen J.T., Houe H., Ersbøll A.K. (2006). Herd-level risk factors for the mortality of cows in Danish dairy herds. Vet. Rec..

[12] Smith J.W., Ely L.O., Chapa A.M. (2000). Effect of region, herd size, and milk production on reasons cows leave the herd. J. Dairy Sci..

[13] Dairy|AHDB. n.d. https://ahdb.org.uk/dairy#.VzDM74RF3ox.

[14] Britt J.H., Cushman R.A., Dechow C.D., Dobson H., Humblot P., Hutjens M.F., Jones G.A., Ruegg P.S., Sheldon I.M., Stevenson J.S. (2018). Invited review: Learning from the future—A vision for dairy farms and cows in 2067. J. Dairy Sci..

[15] Ansari-Lari M., Mohebbi-Fani M., Rowshan-Ghasrodashti A. (2012). Causes of culling in dairy cows and its relation to age at culling and interval from calving in Shiraz, Southern Iran. Vet. Res. Forum.

[16] Nor N.M., Steeneveld W., Hogeveen H. (2014). The average culling rate of Dutch dairy herds over the years 2007 to 2010 and its association with herd reproduction, performance and health. J. Dairy Res..

[17] De Vries A., Olson J.D., Pinedo P.J. (2010). Reproductive risk factors for culling and productive life in large dairy herds in the eastern United States between 2001 and 2006. J. Dairy Sci..

[18] Hadley G.L., Wolf C.A., Harsh S.B. (2006). Dairy cattle culling patterns, explanations, and implications. J. Dairy Sci..

[19] Cha E., Hertl J.A., Schukken Y.H., Tauer L.W., Welcome F.L., Gröhn Y.T. (2013). The effect of repeated episodes of bacteria-specific clinical mastitis on mortality and culling in Holstein dairy cows. J. Dairy Sci..

[20] Cassell B.G., Adamec V., Pearson R.E. (2003). Maternal and fetal inbreeding depression for 70-day nonreturn and calving rate in Holsteins and Jerseys. J. Dairy Sci..

[21] Fabris T.F., Laporta J., Skibiel A.L., Corra F.N., Senn B.D., Wohlgemuth S.E., Dahl G.E. (2019). Effect of heat stress during early, late, and entire dry period on dairy cattle. J. Dairy Sci..

[22] Sewalem A., Miglior F., Kistemaker G.J., Sullivan P., Van Doormaal B.J. (2008). Relationship between reproduction traits and functional longevity in Canadian dairy cattle. J. Dairy Sci..

[23] Ahlman T., Berglund B., Rydhmer L., Strandberg E. (2011). Culling reasons in organic and conventional dairy herds and genotype by environment interaction for longevity. J. Dairy Sci..

[24] Bascom S.S., Young A.J. (1998). A summary of the reasons why farmers cull cows. J. Dairy Sci..

[25] Barbano D.M., Ma Y., Santos M.V. (2006). Influence of raw milk quality on fluid milk shelf life. J. Dairy Sci..

[26] Gröhn Y.T., Eicker S.W., Ducrocq V., Hertl J.A. (1998). Effect of diseases on the culling of Holstein dairy cows in New York State. J. Dairy Sci..

[27] Busanello M., Rossi R.S., Cassoli L.D., Pantoja J.C.F., Machado P.F. (2017). Estimation of prevalence and incidence of subclinical mastitis in a large population of Brazilian dairy herds. J. Dairy Sci..

[28] Ito K., von Keyserlingk M.A.G., LeBlanc S.J., Weary D.M. (2010). Lying behavior as an indicator of lameness in dairy cows. J. Dairy Sci..

[29] Bicalho R.C., Vokey F., Erb H.N., Guard C.L. (2007). Visual locomotion scoring in the first seventy days in milk: Impact on pregnancy and survival. J. Dairy Sci..

[30] Bell M.J., Wall E., Russell G., Roberts D.J., Simm G. (2010). Risk factors for culling in Holstein-Friesian dairy cows. Vet. Rec..

[31] Probo M., Pascottini O.B., LeBlanc S., Opsomer G., Hostens M. (2018). Association between metabolic diseases and the culling risk of high-yielding dairy cows in a transition management facility using survival and decision tree analysis. J. Dairy Sci..

[32] Roberts T., Chapinal N., LeBlanc S.J., Kelton D.F., Dubuc J., Duffield T.F. (2012). Metabolic parameters in transition cows as indicators for early-lactation culling risk. J. Dairy Sci..

[33] Seifi H.A., LeBlanc S.J., Leslie K.E., Duffield T.F. (2011). Metabolic predictors of post-partum disease and culling risk in dairy cattle. Vet. J..

[34] Dürr J.W., Monardes H.G., Cue R.I., Philpot J.C. (1997). Culling in Quebec Holstein herds. Study of phenotypic trends in reasons for disposal. Can. J. Anim. Sci..

[35] Silva L.A.F., Silva E.B., Silva L.M., Trindade B.R., Silva O.C., Romani A.F., Fioravanti M.C.S., Sousa J.N., Franco L.G., Garcia A.M. (2004). Causas de descarte de fêmeas bovinas leiteiras adultas. Rev. Bras. Saúde Produção Anim..

[36] Weigel K.A., Palmer R.W., Caraviello D.Z. (2003). Investigation of factors affecting voluntary and involuntary culling in expanding dairy herds in Wisconsin using survival analysis. J. Dairy Sci..

[37] de Vries A., Steenholdt C., Risco C.A. (2005). Pregnancy rates and milk production in natural service and artificially inseminated dairy herds in Florida and Georgia. J. Dairy Sci..

[38] Rogers G.W., Van Arendonk J.A.M., McDaniel B.T. (1988). Influence of production and prices on optimum culling rates and annualized net revenue. J. Dairy Sci..

[39] Cortez de Souza T., Pinto L.F.B., Cruz V.A.R., Oliveira H.R., Pedrosa V.B., Oliveira G.A., Miglior F., Schenkel F.S., Brito L.F. (2023). A comprehensive characterization of longevity and culling reasons in Canadian Holstein cattle based on various systematic factors. Transl. Anim. Sci..

[40] Tao S., Dahl G.E., Laporta J., Bernard J.K., Orellana R.M., Marins T.N. (2019). Physiology Symposium: Effects of heat stress during late gestation on the dam and its calf. J. Anim. Sci..

[41] McConnel C.S., Lombard J.E., Wagner B.A., Garry F.B. (2008). Evaluation of factors associated with increased dairy cow mortality on United States dairy operations. J. Dairy Sci..

